# Assessment of Sleep Habits and Problems in Children Aged 7 to 10: An Observational Study.

**DOI:** 10.1192/j.eurpsy.2024.934

**Published:** 2024-08-27

**Authors:** J. Camilo

**Affiliations:** Psychiatry, CHTMAD, Vila Real, Portugal

## Abstract

**Introduction:**

Appropriate sleep habits play a pivotal role in the physical and psychological development of children. However, sleep deprivation or sleep problems can have a significant impact on children’s mental health and daily functioning. This study investigates the sleep habits and problems in children aged 7 to 10 who attend the Child and Adolescent Psychiatry Service at CHTMAD, Vila Real, Portugal.

**Objectives:**

The primary objective of this study is to assess the sleep habits and problems in children aged 7 to 10, aiming to establish data that can guide the development, implementation, and reevaluation of future interventions tailored to this age group.

**Methods:**

This study is observational in nature and involved the participation of 21 patients from the Child and Adolescent Psychiatry Service at CHTMAD, Vila Real, Portugal, throughout the year 2022. Questionnaires related to sleep habits were administered to this population. Parents were invited to complete the Children’s Sleep Habit Questionnaire (adapted from the Children’s Sleep Habit Questionnaire by Prof. Owens, 2000), while the children (patients) were asked to fill out the Sleep Self Report-PT (adapted from Owens 2000 Research Version) and the Strengths and Difficulties Questionnaire (SDQ-Por, by Robert Goodman, 2005).

**Results:**

As of now, the results of this study are still being processed. The data collected from the questionnaires will be analyzed to gain insights into the sleep habits and issues of children aged 7 to 10 years attending the Child and Adolescent Psychiatry Service in Vila Real, Portugal. Findings will be discussed, and any significant observations or trends will be highlighted.

**Conclusions:**

This research aims to provide valuable insights into the sleep patterns and problems experienced by children in the specified age group. By understanding these issues, we can develop and implement targeted interventions to improve sleep quality and overall mental well-being. The conclusions drawn from this study will contribute to the development of evidence-based strategies for enhancing the sleep health of children in the Child and Adolescent Psychiatry Service at CHTMAD, Vila Real, Portugal.

**Disclosure of Interest:**

None Declared

